# Supplementary material for: How Effective Is Phage Therapy for Prosthetic Joint Infections? A Preliminary Systematic Review and Proportional Meta-Analysis of Early Outcomes
Source: Medicina (Kaunas). 2024 May 9;60(5):790. doi: 10.3390/medicina60050790 (PMC11122905; doi:10.3390/medicina60050790)
Supplement: Supplementary file 1 [file medicina-60-00790-s001.zip › S4.pdf]

Data Extraction Tool

Study ID:
Title:
Lead author contact details:
Country in which the study conducted:

1. United States
2. UK
3. Canada
4. Australia
5. Other

Study Purpose:

Study design:
Start date:
End date:
Study funding sources:

Participants
Population description:
Setting:
Inclusion criteria:
Exclusion criteria:
Total number of participants who received therapy:
Baseline Population Characteristics:

	Age	Gender	Presenting Problem	Organism(s) causing infection	Prior treatment failure with standard of care?	Therapeutic Dead end? (IE facing amputation if not PT)
**Patient**						

Intervention and Comparisons, Outcomes, and Complications

	Phage(s) delivered	Delivery mechanism (IV vs oral vs intraarticular vs combined IV+Intraarticular vs other)	Phage Dosing	Phage therapy duration	Delivered with concomitant abx?	Concomitant debridement?	Delivered during or after debridement and implant retention?	Delivered after 1 stage revision?	Delivered after 2 stage revision?	Length of follow-up	Any documented complications?
**Patient**											

	Number	Proportion (out of total patients recieving therapy)
**Number demonstrating infection eradication (clinical criteria alone)**		
**Number demonstrating infection eradication (arthrocentesis/ culture data supporting negative growth)**		
**Number demonstrating infection eradication (PET Scan supporing)**		
**Total number with infection eradication**		
