# Supplementary material for: How Effective Is Phage Therapy for Prosthetic Joint Infections? A Preliminary Systematic Review and Proportional Meta-Analysis of Early Outcomes
Source: Medicina (Kaunas). 2024 May 9;60(5):790. doi: 10.3390/medicina60050790 (PMC11122905; doi:10.3390/medicina60050790)
Supplement: Supplementary file 1 [file medicina-60-00790-s001.zip › S5.pdf]

Supplemental File S5: Consensus Quality Assessment Evaluation of Included Studies

Quality Assessment for Case Series Using the NHLBI Assessment Tool

	Clear Study Objectives	Clear Study Population Description	Consecutive Cases	Comparable Subjects	Clearly Defined Intervention	Clearly Defined Outcomes	Adequate Follow-up	Clearly Described Statistics	Well Described Results	Quality Rating (Good, Fair, or Poor)
**Doub et al. 2023 [25]**			NR*							Fair
**Ferry et al. 2020 [26]**										Fair
**Onallah et al. 2023 [19]**			NR*							Poor

*NR = Not Reported. Green = Meets criteria. Red = Does not meet criteria.

Quality Assessment for Case Control Studies Using the NHLBI Assessment Tool

	Clear Study Objectives	Clear Study Population Description	Participation > 50%	Comparable subjects	Sample Size Justification Provided	Exposure Measure Prior to Outcome	Sufficient Time Frame	Varied Categories of the Exposure Measured, if Applicable	Exposures clearly defined	Exposure Measured More than Once Over Time	Outcomes Clearly Defined	Assessors Blinded to Exposure	Loss to Follow-up >20%	Potential Confounders Measured and Adjusted	Quality Rating (Good, Fair, or Poor)
**Fedorov et al. 2023 [29]**	Green	Green	Green	Red	Green	Green	Green	N/A	Green	Green	Green	NR*	Green	Red	Fair

*NR = Not Reported. Green = Meets criteria. Red = Does not meet criteria.
